# Crystal structure of (2*Z*,5*Z*)-3-(4-meth­oxy­phen­yl)-2-[(4-meth­oxy­phenyl)­imino]-5-[(*E*)-3-(2-nitro­phen­yl)allyl­idene]-1,3-thia­zolidin-4-one

**DOI:** 10.1107/S2056989016000207

**Published:** 2016-01-13

**Authors:** Rachida Rahmani, Ahmed Djafri, Jean-Claude Daran, Ayada Djafri, Abdelkader Chouaih, Fodil Hamzaoui

**Affiliations:** a Laboratory of Technology and Properties of Solids, Abdelhamid Ibn Badis University, BP 227 Mostaganem 27000, Algeria; b Laboratoire de Chimie de Coordination, 205 route de Narbonne, 31077 Toulouse Cedex, France; c Laboratoire de Synthèse Organique Appliquée (LSOA), Département de Chimie, Faculté de Sciences, University of Oran Es-Sénia, 31000 Oran, Algeria

**Keywords:** crystal structure, thia­zolidinone, hydrogen bonding, π–π stacking

## Abstract

In the title compound, C_26_H_21_N_3_O_5_S, the thia­zole ring is nearly planar with a maximum deviation of 0.017 (2) Å, and is twisted with respect to the three benzene rings, making dihedral angles of 25.52 (12), 85.77 (12) and 81.85 (13)°. In the crystal, weak C—H⋯O hydrogen bonds and C—H⋯π inter­actions link the mol­ecules into a three-dimensional supra­molecular architecture. Aromatic π–π stacking is also observed between the parallel nitro­benzene rings of neighbouring mol­ecules, the centroid-to-centroid distance being 3.5872 (15) Å.

## Abstract 2

The thia­zole ring of the title compound is twisted with respect to the three benzene rings, making dihedral angles of 25.52 (12), 85.77 (12) and 81.85 (13)°.

## Chemical context   

Heterocycles containing a thia­zole ring are found to exhibit a wide spectrum of biological activities (Gautam *et al.*, 2015 ▸; Asif, 2015 ▸; Abhinit *et al.*, 2009 ▸). The thia­zolidinones that are used widely in medication are derived from thia­zolidines containing sulfur and nitro­gen in a five-membered ring (Meera *et al.*, 2014 ▸; Nowaczyk *et al.*, 2014 ▸; Toubal *et al.*, 2012 ▸). Knowledge of the crystal structures of these compounds is crucial for understanding the related biological phenomena (Singh *et al.*, 1981 ▸; Ameta *et al.*, 2014 ▸; Gouda *et al.*, 2011 ▸). As part of our studies in this area, we herein report the synthesis and crystal structure of the title compound.
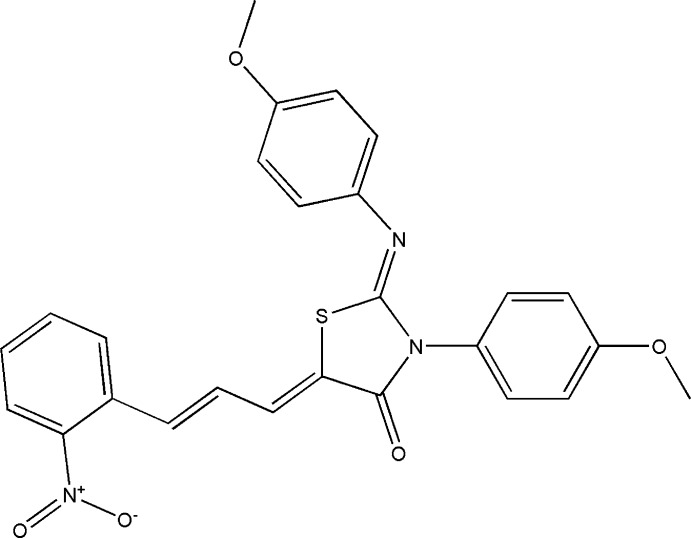


## Structural commentary   

**Figure 1:**
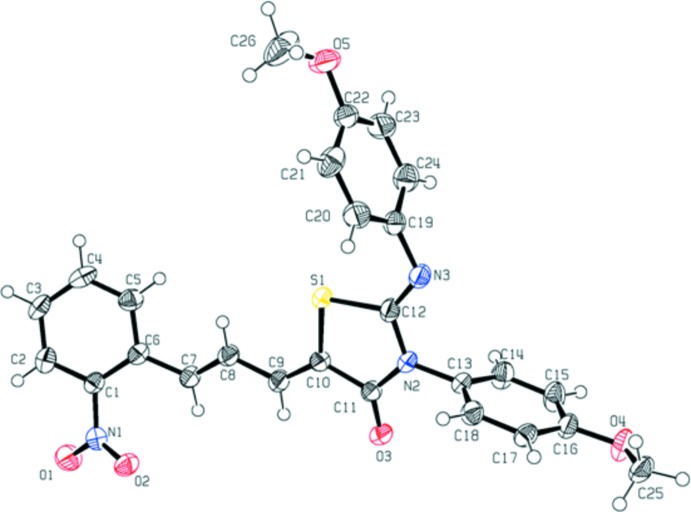
The mol­ecular structure of the title compound showing the atom-numbering scheme. Displacement ellipsoids are drawn at the 50% probability level.

The mol­ecular structure with atomic numbering scheme for the title compound is given in Fig. 1 ▸. The N2—C11 and N2—C12 bond lengths [1.385 (3) and 1.389 (3) Å] are inter­mediate between the classical C—N single-bond length (1.47 Å) and C=N double-bond length (1.27 Å) (Bhagavan, 2002 ▸), indicating that the thia­zole moiety is an effective electron-conjugated substructure. The C—S bond lengths in the thia­zol rings [S1—C10 = 1.753 (3) and S1—C12 = 1.777 (2) Å] are consistant with the normal C*sp* ^2^—S single bond length of 1.76 Å (Sarkar *et al.*, 1984 ▸). The C16—O4 bond length [1.365 (3) Å] and C22—O5 bond length [1.375 (3) Å] are notably shorter than the normal O—C single bond (1.427 Å) (Rong Wan *et al.*, 2008 ▸), indicating that the *p* orbital occupied lone pair electrons of the oxygen atom in CH_3_O and the π orbital in the benzene ring has *p*–π conjugation. The shorter bond length of C26—O5 [1.385 (5) Å] might be also caused by the delocalized electron density of the conjugated benzene ring. The C25—O4 [1.431 (3) Å] bond length is normal for a C—O single bond.

The thia­zole ring is nearly planar with a maximum deviation of 0.017 (2) Å, and is twisted with respect to the three benzene rings, making dihedral angles of 25.52 (12), 85.77 (12) and 81.85 (13)°with the C1–C6, C13–C18 and C19–C24 rings, respectively.

## Supra­molecular features   

**Figure 2:**
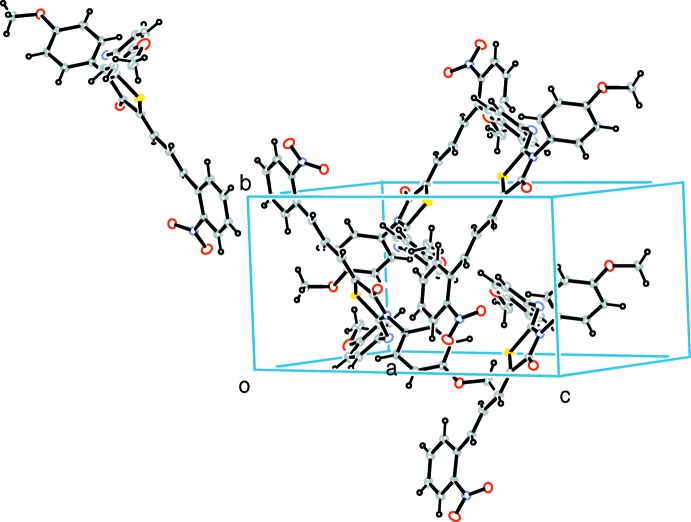
The crystal packing diagram showing π–π stacking between the nitro­benzene rings of the neighbouring mol­ecules.

In the crystal, weak C—H⋯O hydrogen bonds and C—H⋯π inter­actions (Table 1 ▸, Fig. 2 ▸) link the mol­ecules into a three-dimensional supra­molecular architecture. π–π stacking is also observed between the nearly parallel benzene rings of neighbouring mol­ecules, the centroid-to-centroid distance being 3.5872 (15) Å.

## Synthesis and crystallization   

**Figure 3:**
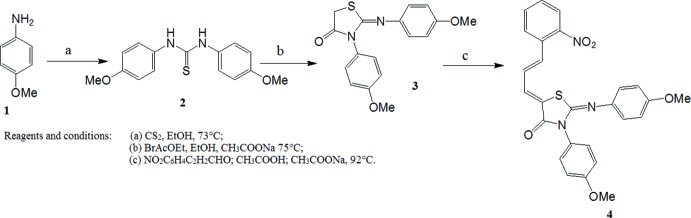
Chemical pathway showing the formation of the title compound.

The synthesis of the title compound was performed according to the scheme in Fig. 3 ▸. To a solution of **3** (0.01 mol) in 10 mL of acetic acid and three equivalents of anhydrous sodium acetate was added 2-nitro­phenyl­cinamaldehyde (0.01 mol). The mixture was heated at reflux with stirring, using CH_2_Cl_2_ (20 mL) for 4 h. The reaction was monitored by TLC using CH_2_Cl_2_/CH_3_CO_2_C_2_H_5_ (9/1) as solvent system. The separated solid was filtered, washed with cold water and dried to give a yellow solid with a moderate yield 75% and melting point 484 K. Single crystals of the title compound suitable for X-ray diffraction were obtained from an ethanol solution.

IR (KBr, cm^−1^): 3423.03, 2951 (C—H), 1712 (C=O), 1640.16 (C=N), 1509.93 (C=C), 1030 (C—N), 741(C—S). ^1^H NMR, (CDCl_3_, 300 MHz) δ (p.p.m.) *J* (Hz): 3.81 (*s*, 3H, OCH_3_), 3.85 (*s*, 3H, OCH_3_), 6.71 (*dd*, 1H, *J* = 15.0 Hz, *J* = 11.55 Hz, CH), 6.90 (*s*, 4H, Ar-H), 7.04 (*d*, 2H, *J* = 8.8 Hz, Ar-H), 7.35 (*d*, 2H, *J* = 8.8 Hz, Ar-H), 7.43–7.67 (*m*, 5H, Ar-H), 8.0 (*d*, 1H, *J* = 8.72 Hz, Chet=CH). ^13^C NMR, (CDCl_3_, 300 MHz) δ (p.p.m.): 55.57 (O—CH_3_), 55.65 (O—CH_3_), 114.57, 114.85, 122.34, 125.22, 126.37, 127.35, 127.99, 128.50, 129.20, 129.57, 129.60, 131.61, 133.36, 135.79, 141.83, 148.13, 150.72, 157.20 (Chet=C), 159.90 (C=N), 165.87 (C=O).

## Refinement   

Crystal data, data collection and structure refinement details are summarized in Table 2 ▸. H atoms in the title compound were placed in calculated positions (C—H = 0.96–1.08 Å) and allowed to ride on their parent atoms with *U*_iso_(H) = 1.5*U*_eq_(C) for methyl H atoms and 1.2*U*_eq_(C) for other H atoms.

## Supplementary Material

Crystal structure: contains datablock(s) I, global. DOI: 10.1107/S2056989016000207/xu5881sup1.cif

Structure factors: contains datablock(s) I. DOI: 10.1107/S2056989016000207/xu5881Isup2.hkl

CCDC reference: 1402626

Additional supporting information:  crystallographic information; 3D view; checkCIF report

## Figures and Tables

**Table 1:** Hydrogen-bond geometry (Å, °) *Cg*3 is the centroid of the C13–C18 ring.

*D*—H⋯*A*	*D*—H	H⋯*A*	*D*⋯*A*	*D*—H⋯*A*
C7—H7⋯O3^i^	1.00 (2)	2.55 (2)	3.197 (3)	122 (1)
C9—H9⋯O2^ii^	0.97 (2)	2.58 (2)	3.400 (3)	142 (1)
C15—H15⋯O1^iii^	0.93	2.59	3.286 (3)	132
C3—H3⋯*Cg*3^iv^	0.93	2.80	3.560 (3)	140

Symmetry codes: (i) 
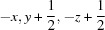
; (ii) 
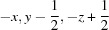
; (iii) 
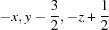
; (iv) 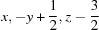.

**Table 2:** Experimental details

Crystal data	
Chemical formula	C_26_H_21_N_3_O_5_S
*M*_r_	487.52
Crystal system, space group	Monoclinic, *P*2_1_/*c*
Temperature (K)	173
*a*, *b*, *c* (Å)	13.2727 (10), 8.6401 (4), 21.3018 (12)
β (°)	105.316 (7)
*V* (Å^3^)	2356.1 (3)
*Z*	4
Radiation type	Mo *K*α
μ (mm^−1^)	0.18
Crystal size (mm)	0.25 × 0.21 × 0.12

Data collection	
Diffractometer	Nonius Kappa CCD
Absorption correction	ψ scan (North *et al.*, 1968 ▸)
*T*_min_, *T*_max_	0.856, 0.919
No. of measured, independent and observed [*I* > 2σ(*I*)] reflections	26882, 5954, 3690
*R*_int_	0.062
(sin θ/λ)_max_ (Å^−1^)	0.692

Refinement	
*R*[*F* ^2^ > 2σ(*F* ^2^)], *wR*(*F* ^2^), *S*	0.064, 0.166, 1.02
No. of reflections	5954
No. of parameters	322
H-atom treatment	H-atom parameters not refined
Δρ_max_, Δρ_min_ (e Å^−3^)	0.49, −0.34

Computer programs: *KappaCCD* (Nonius, 1998 ▸), *DENZO* and *SCALEPACK* (Otwinowski & Minor, 1997 ▸), *SHELXS97* (Sheldrick, 2008 ▸), *SHELXL2014*/7 (Sheldrick, 2015 ▸), *ORTEP-3 for Windows* (Farrugia, 2012 ▸), and *Mercury* (Macrae *et al.*, 2006 ▸).

## supplementary crystallographic information

### Crystal data

**Table:**

C_26_H_21_N_3_O_5_S	*F*(000) = 1016
*M**_r_* = 487.52	*D*_x_ = 1.374 Mg m^−^^3^
Monoclinic, *P*2_1_/*c*	Mo *K*α radiation, λ = 0.71073 Å
*a* = 13.2727 (10) Å	Cell parameters from 100 reflections
*b* = 8.6401 (4) Å	θ = 2–29°
*c* = 21.3018 (12) Å	µ = 0.18 mm^−^^1^
β = 105.316 (7)°	*T* = 173 K
*V* = 2356.1 (3) Å^3^	Prism, yellow
*Z* = 4	0.25 × 0.21 × 0.12 mm

### Data collection

**Table:**

Nonius Kappa CCD diffractometer	3690 reflections with *I* > 2σ(*I*)
θ/2θ scans	*R*_int_ = 0.062
Absorption correction: ψ scan (North *et al.*, 1968)	θ_max_ = 29.5°, θ_min_ = 2.9°
*T*_min_ = 0.856, *T*_max_ = 0.919	*h* = −17→17
26882 measured reflections	*k* = −11→11
5954 independent reflections	*l* = −29→27

### Refinement

**Table:**

Refinement on *F*^2^	0 restraints
Least-squares matrix: full	Hydrogen site location: inferred from neighbouring sites
*R*[*F*^2^ > 2σ(*F*^2^)] = 0.064	H-atom parameters not refined
*wR*(*F*^2^) = 0.166	*w* = 1/[σ^2^(*F*_o_^2^) + (0.0731*P*)^2^ + 0.7139*P*] where *P* = (*F*_o_^2^ + 2*F*_c_^2^)/3
*S* = 1.02	(Δ/σ)_max_ < 0.001
5954 reflections	Δρ_max_ = 0.49 e Å^−^^3^
322 parameters	Δρ_min_ = −0.34 e Å^−^^3^

### Special details

**Table:**

Geometry. All e.s.d.'s (except the e.s.d. in the dihedral angle between two l.s. planes) are estimated using the full covariance matrix. The cell e.s.d.'s are taken into account individually in the estimation of e.s.d.'s in distances, angles and torsion angles; correlations between e.s.d.'s in cell parameters are only used when they are defined by crystal symmetry. An approximate (isotropic) treatment of cell e.s.d.'s is used for estimating e.s.d.'s involving l.s. planes.

### Fractional atomic coordinates and isotropic or equivalent isotropic displacement parameters (Å^2^)

**Table:**

	*x*	*y*	*z*	*U*_iso_*/*U*_eq_	
S1	0.28022 (6)	0.41002 (7)	0.22265 (3)	0.0317 (2)	
O3	0.14135 (16)	0.4595 (2)	0.35874 (8)	0.0356 (5)	
O4	0.29886 (17)	−0.0889 (2)	0.54702 (9)	0.0448 (5)	
O2	0.08864 (16)	1.1504 (2)	0.22010 (8)	0.0389 (5)	
N2	0.25988 (18)	0.2956 (2)	0.33219 (9)	0.0294 (5)	
O1	−0.00470 (18)	1.3059 (2)	0.14901 (10)	0.0498 (6)	
N3	0.37138 (19)	0.1521 (2)	0.28522 (10)	0.0345 (5)	
N1	0.05420 (18)	1.1947 (2)	0.16354 (11)	0.0335 (5)	
C11	0.1930 (2)	0.4215 (3)	0.32243 (12)	0.0287 (6)	
C18	0.3513 (2)	0.2234 (3)	0.44293 (12)	0.0320 (6)	
H18	0.3978	0.3040	0.4433	0.038*	
C13	0.2708 (2)	0.1963 (3)	0.38783 (11)	0.0283 (6)	
C10	0.1968 (2)	0.5044 (3)	0.26177 (12)	0.0295 (6)	
C1	0.0854 (2)	1.1147 (3)	0.11192 (11)	0.0277 (6)	
C8	0.1515 (2)	0.7251 (3)	0.18670 (12)	0.0326 (6)	
H8	0.1950 (14)	0.6898 (12)	0.1613 (8)	0.039*	
C19	0.4160 (2)	0.1407 (3)	0.23137 (12)	0.0335 (6)	
C17	0.3631 (2)	0.1318 (3)	0.49738 (12)	0.0295 (6)	
H17	0.4172	0.1505	0.5344	0.035*	
C6	0.1071 (2)	0.9552 (3)	0.11536 (11)	0.0290 (6)	
C9	0.1448 (2)	0.6358 (3)	0.24244 (12)	0.0324 (6)	
H9	0.0994 (15)	0.6736 (12)	0.2677 (8)	0.039*	
O5	0.5431 (2)	0.0888 (3)	0.07390 (10)	0.0618 (7)	
C7	0.0979 (2)	0.8569 (3)	0.16964 (12)	0.0304 (6)	
H7	0.0486 (15)	0.8903 (10)	0.1956 (8)	0.037*	
C12	0.3111 (2)	0.2662 (3)	0.28442 (11)	0.0300 (6)	
C16	0.2929 (2)	0.0105 (3)	0.49635 (12)	0.0325 (6)	
C2	0.0961 (2)	1.2058 (3)	0.06006 (12)	0.0370 (7)	
H2	0.0823	1.3114	0.0597	0.044*	
C14	0.2003 (2)	0.0780 (3)	0.38674 (13)	0.0396 (7)	
H14	0.1461	0.0606	0.3496	0.047*	
C5	0.1365 (2)	0.8925 (3)	0.06228 (12)	0.0363 (6)	
H5	0.1494	0.7867	0.0618	0.044*	
C3	0.1275 (2)	1.1394 (3)	0.00918 (12)	0.0368 (7)	
H3	0.1354	1.1995	−0.0254	0.044*	
C4	0.1471 (2)	0.9813 (4)	0.01070 (13)	0.0412 (7)	
H4	0.1675	0.9350	−0.0234	0.049*	
C25	0.3914 (2)	−0.0808 (3)	0.59982 (12)	0.0404 (7)	
H25A	0.4516	−0.0953	0.5835	0.061*	
H25B	0.3894	−0.1603	0.6310	0.061*	
H25C	0.3952	0.0187	0.6204	0.061*	
C24	0.3733 (2)	0.0413 (3)	0.18017 (14)	0.0422 (7)	
H24	0.3152	−0.0181	0.1807	0.051*	
C21	0.5481 (2)	0.2158 (4)	0.17753 (14)	0.0438 (7)	
H21	0.6056	0.2763	0.1764	0.053*	
C22	0.5053 (3)	0.1118 (3)	0.12736 (13)	0.0414 (7)	
C15	0.2106 (2)	−0.0143 (3)	0.44110 (13)	0.0424 (7)	
H15	0.1627	−0.0930	0.4408	0.051*	
C20	0.5029 (2)	0.2266 (3)	0.22887 (14)	0.0410 (7)	
H20	0.5319	0.2939	0.2629	0.049*	
C23	0.4174 (3)	0.0305 (3)	0.12806 (13)	0.0457 (8)	
H23	0.3865	−0.0331	0.0931	0.055*	
C26	0.6246 (3)	0.1806 (5)	0.06639 (18)	0.0720 (12)	
H26A	0.6056	0.2876	0.0670	0.108*	
H26B	0.6391	0.1571	0.0256	0.108*	
H26C	0.6857	0.1606	0.1013	0.108*	

### Atomic displacement parameters (Å^2^)

**Table:**

	*U*^11^	*U*^22^	*U*^33^	*U*^12^	*U*^13^	*U*^23^
S1	0.0421 (4)	0.0288 (3)	0.0284 (3)	0.0034 (3)	0.0165 (3)	0.0052 (3)
O3	0.0422 (12)	0.0378 (10)	0.0313 (10)	0.0025 (8)	0.0177 (9)	0.0018 (8)
O4	0.0505 (14)	0.0465 (11)	0.0335 (10)	−0.0113 (10)	0.0043 (10)	0.0158 (9)
O2	0.0459 (13)	0.0443 (10)	0.0261 (10)	−0.0015 (9)	0.0085 (9)	0.0007 (8)
N2	0.0369 (13)	0.0303 (10)	0.0229 (10)	0.0013 (9)	0.0111 (9)	0.0047 (8)
O1	0.0568 (15)	0.0421 (11)	0.0519 (12)	0.0192 (10)	0.0171 (11)	0.0056 (9)
N3	0.0391 (14)	0.0349 (11)	0.0330 (12)	0.0076 (10)	0.0156 (10)	0.0088 (9)
N1	0.0333 (14)	0.0305 (11)	0.0363 (13)	−0.0008 (10)	0.0083 (10)	0.0018 (9)
C11	0.0331 (15)	0.0287 (12)	0.0250 (12)	−0.0039 (11)	0.0094 (11)	−0.0006 (10)
C18	0.0321 (16)	0.0310 (13)	0.0339 (14)	−0.0072 (11)	0.0108 (12)	0.0003 (10)
C13	0.0353 (15)	0.0286 (12)	0.0228 (12)	0.0006 (11)	0.0108 (11)	0.0028 (9)
C10	0.0336 (15)	0.0283 (12)	0.0282 (13)	−0.0023 (11)	0.0113 (11)	−0.0012 (10)
C1	0.0249 (14)	0.0339 (13)	0.0228 (12)	−0.0027 (10)	0.0033 (10)	0.0022 (10)
C8	0.0355 (16)	0.0331 (13)	0.0323 (14)	0.0046 (11)	0.0145 (12)	0.0038 (11)
C19	0.0382 (17)	0.0325 (13)	0.0287 (14)	0.0069 (12)	0.0070 (12)	0.0057 (11)
C17	0.0277 (14)	0.0369 (13)	0.0220 (12)	−0.0031 (11)	0.0029 (10)	0.0009 (10)
C6	0.0246 (14)	0.0360 (13)	0.0262 (13)	0.0024 (11)	0.0066 (11)	0.0043 (10)
C9	0.0350 (16)	0.0328 (13)	0.0320 (14)	0.0012 (11)	0.0136 (12)	0.0034 (11)
O5	0.089 (2)	0.0627 (14)	0.0466 (13)	0.0304 (14)	0.0413 (13)	0.0104 (11)
C7	0.0301 (15)	0.0332 (13)	0.0292 (13)	0.0020 (11)	0.0099 (11)	0.0042 (10)
C12	0.0347 (16)	0.0289 (12)	0.0251 (13)	−0.0029 (11)	0.0057 (11)	0.0043 (10)
C16	0.0380 (16)	0.0322 (13)	0.0267 (13)	−0.0032 (11)	0.0076 (12)	0.0060 (10)
C2	0.0367 (17)	0.0378 (14)	0.0358 (15)	−0.0016 (12)	0.0083 (13)	0.0088 (12)
C14	0.0439 (18)	0.0400 (14)	0.0280 (14)	−0.0110 (13)	−0.0023 (12)	0.0029 (11)
C5	0.0344 (16)	0.0417 (15)	0.0334 (14)	0.0056 (12)	0.0102 (12)	−0.0001 (11)
C3	0.0363 (17)	0.0498 (16)	0.0250 (13)	−0.0067 (13)	0.0095 (12)	0.0083 (12)
C4	0.0393 (18)	0.0611 (18)	0.0259 (14)	0.0010 (14)	0.0134 (12)	−0.0010 (13)
C25	0.0487 (19)	0.0479 (16)	0.0231 (13)	0.0058 (14)	0.0069 (13)	0.0086 (11)
C24	0.0420 (18)	0.0426 (15)	0.0404 (16)	−0.0018 (13)	0.0081 (14)	0.0027 (13)
C21	0.0327 (17)	0.0575 (18)	0.0430 (17)	0.0041 (14)	0.0133 (14)	0.0133 (14)
C22	0.055 (2)	0.0394 (15)	0.0338 (15)	0.0207 (14)	0.0188 (14)	0.0103 (12)
C15	0.0464 (19)	0.0370 (14)	0.0399 (16)	−0.0170 (13)	0.0044 (14)	0.0069 (12)
C20	0.0431 (18)	0.0414 (15)	0.0373 (16)	−0.0006 (13)	0.0086 (14)	−0.0011 (12)
C23	0.059 (2)	0.0449 (16)	0.0297 (15)	−0.0013 (15)	0.0048 (14)	−0.0069 (12)
C26	0.065 (3)	0.100 (3)	0.065 (2)	0.041 (2)	0.042 (2)	0.040 (2)

### Geometric parameters (Å, º)

**Table:**

S1—C10	1.753 (3)	C9—H9	0.96 (3)
S1—C12	1.777 (2)	O5—C22	1.375 (3)
O3—C11	1.206 (3)	O5—C26	1.385 (5)
O4—C16	1.365 (3)	C7—H7	1.00 (3)
O4—C25	1.431 (3)	C16—C15	1.395 (4)
O2—N1	1.232 (3)	C2—C3	1.384 (4)
N2—C11	1.385 (3)	C2—H2	0.9300
N2—C12	1.389 (3)	C14—C15	1.383 (4)
N2—C13	1.439 (3)	C14—H14	0.9300
O1—N1	1.226 (3)	C5—C4	1.377 (4)
N3—C12	1.267 (3)	C5—H5	0.9300
N3—C19	1.426 (3)	C3—C4	1.389 (4)
N1—C1	1.449 (3)	C3—H3	0.9300
C11—C10	1.490 (3)	C4—H4	0.9300
C18—C17	1.378 (3)	C25—H25A	0.9600
C18—C13	1.383 (4)	C25—H25B	0.9600
C18—H18	0.9300	C25—H25C	0.9600
C13—C14	1.382 (4)	C24—C23	1.388 (4)
C10—C9	1.336 (4)	C24—H24	0.9300
C1—C2	1.394 (3)	C21—C20	1.383 (4)
C1—C6	1.406 (3)	C21—C22	1.397 (4)
C8—C7	1.341 (3)	C21—H21	0.9300
C8—C9	1.438 (3)	C22—C23	1.365 (4)
C8—H8	0.94 (3)	C15—H15	0.9300
C19—C20	1.385 (4)	C20—H20	0.9300
C19—C24	1.386 (4)	C23—H23	0.9300
C17—C16	1.399 (4)	C26—H26A	0.9600
C17—H17	0.9300	C26—H26B	0.9600
C6—C5	1.399 (4)	C26—H26C	0.9600
C6—C7	1.465 (3)		
			
C10—S1—C12	91.41 (12)	O4—C16—C17	124.0 (2)
C16—O4—C25	116.7 (2)	C15—C16—C17	119.9 (2)
C11—N2—C12	116.9 (2)	C3—C2—C1	120.1 (3)
C11—N2—C13	120.9 (2)	C3—C2—H2	120.0
C12—N2—C13	122.1 (2)	C1—C2—H2	120.0
C12—N3—C19	116.0 (2)	C13—C14—C15	119.7 (2)
O1—N1—O2	122.5 (2)	C13—C14—H14	120.1
O1—N1—C1	118.3 (2)	C15—C14—H14	120.1
O2—N1—C1	119.2 (2)	C4—C5—C6	122.5 (3)
O3—C11—N2	124.6 (2)	C4—C5—H5	118.7
O3—C11—C10	125.5 (2)	C6—C5—H5	118.7
N2—C11—C10	109.9 (2)	C2—C3—C4	118.9 (2)
C17—C18—C13	120.5 (2)	C2—C3—H3	120.6
C17—C18—H18	119.7	C4—C3—H3	120.6
C13—C18—H18	119.8	C5—C4—C3	120.6 (3)
C14—C13—C18	120.5 (2)	C5—C4—H4	119.7
C14—C13—N2	120.5 (2)	C3—C4—H4	119.7
C18—C13—N2	119.0 (2)	O4—C25—H25A	109.5
C9—C10—C11	122.8 (2)	O4—C25—H25B	109.5
C9—C10—S1	126.1 (2)	H25A—C25—H25B	109.5
C11—C10—S1	111.07 (18)	O4—C25—H25C	109.5
C2—C1—C6	122.2 (2)	H25A—C25—H25C	109.5
C2—C1—N1	116.2 (2)	H25B—C25—H25C	109.5
C6—C1—N1	121.6 (2)	C19—C24—C23	120.0 (3)
C7—C8—C9	122.4 (3)	C19—C24—H24	120.0
C7—C8—H8	118.8	C23—C24—H24	120.0
C9—C8—H8	118.8	C20—C21—C22	118.3 (3)
C20—C19—C24	118.2 (3)	C20—C21—H21	120.8
C20—C19—N3	121.4 (2)	C22—C21—H21	120.8
C24—C19—N3	120.4 (3)	C23—C22—O5	115.8 (3)
C18—C17—C16	119.3 (2)	C23—C22—C21	119.9 (3)
C18—C17—H17	120.3	O5—C22—C21	124.3 (3)
C16—C17—H17	120.3	C14—C15—C16	120.0 (2)
C5—C6—C1	115.8 (2)	C14—C15—H15	120.0
C5—C6—C7	120.8 (2)	C16—C15—H15	120.0
C1—C6—C7	123.4 (2)	C21—C20—C19	122.3 (3)
C10—C9—C8	124.8 (3)	C21—C20—H20	118.8
C10—C9—H9	117.6	C19—C20—H20	118.8
C8—C9—H9	117.6	C22—C23—C24	121.1 (3)
C22—O5—C26	118.7 (3)	C22—C23—H23	119.5
C8—C7—C6	123.9 (3)	C24—C23—H23	119.5
C8—C7—H7	118.1	O5—C26—H26A	109.5
C6—C7—H7	118.0	O5—C26—H26B	109.5
N3—C12—N2	124.2 (2)	H26A—C26—H26B	109.5
N3—C12—S1	125.1 (2)	O5—C26—H26C	109.5
N2—C12—S1	110.65 (18)	H26A—C26—H26C	109.5
O4—C16—C15	116.1 (2)	H26B—C26—H26C	109.5
			
C12—N2—C11—O3	178.2 (2)	C11—N2—C12—N3	−177.1 (2)
C13—N2—C11—O3	1.4 (4)	C13—N2—C12—N3	−0.3 (4)
C12—N2—C11—C10	−3.3 (3)	C11—N2—C12—S1	2.9 (3)
C13—N2—C11—C10	179.8 (2)	C13—N2—C12—S1	179.67 (18)
C17—C18—C13—C14	0.7 (4)	C10—S1—C12—N3	178.9 (2)
C17—C18—C13—N2	179.1 (2)	C10—S1—C12—N2	−1.16 (19)
C11—N2—C13—C14	83.3 (3)	C25—O4—C16—C15	170.5 (3)
C12—N2—C13—C14	−93.4 (3)	C25—O4—C16—C17	−9.6 (4)
C11—N2—C13—C18	−95.1 (3)	C18—C17—C16—O4	178.6 (3)
C12—N2—C13—C18	88.2 (3)	C18—C17—C16—C15	−1.5 (4)
O3—C11—C10—C9	2.6 (4)	C6—C1—C2—C3	1.1 (4)
N2—C11—C10—C9	−175.9 (2)	N1—C1—C2—C3	178.8 (2)
O3—C11—C10—S1	−179.3 (2)	C18—C13—C14—C15	−0.3 (4)
N2—C11—C10—S1	2.3 (3)	N2—C13—C14—C15	−178.7 (3)
C12—S1—C10—C9	177.4 (3)	C1—C6—C5—C4	2.1 (4)
C12—S1—C10—C11	−0.63 (19)	C7—C6—C5—C4	−179.2 (3)
O1—N1—C1—C2	33.4 (4)	C1—C2—C3—C4	0.4 (4)
O2—N1—C1—C2	−145.6 (2)	C6—C5—C4—C3	−0.7 (5)
O1—N1—C1—C6	−148.8 (2)	C2—C3—C4—C5	−0.7 (4)
O2—N1—C1—C6	32.2 (4)	C20—C19—C24—C23	−0.4 (4)
C12—N3—C19—C20	81.3 (3)	N3—C19—C24—C23	−179.8 (2)
C12—N3—C19—C24	−99.3 (3)	C26—O5—C22—C23	172.6 (3)
C13—C18—C17—C16	0.2 (4)	C26—O5—C22—C21	−4.3 (4)
C2—C1—C6—C5	−2.3 (4)	C20—C21—C22—C23	3.7 (4)
N1—C1—C6—C5	−179.9 (2)	C20—C21—C22—O5	−179.4 (3)
C2—C1—C6—C7	179.1 (2)	C13—C14—C15—C16	−1.0 (5)
N1—C1—C6—C7	1.5 (4)	O4—C16—C15—C14	−178.1 (3)
C11—C10—C9—C8	175.7 (2)	C17—C16—C15—C14	1.9 (5)
S1—C10—C9—C8	−2.1 (4)	C22—C21—C20—C19	−1.4 (4)
C7—C8—C9—C10	−179.6 (3)	C24—C19—C20—C21	−0.3 (4)
C9—C8—C7—C6	176.6 (2)	N3—C19—C20—C21	179.2 (2)
C5—C6—C7—C8	26.7 (4)	O5—C22—C23—C24	178.5 (3)
C1—C6—C7—C8	−154.7 (3)	C21—C22—C23—C24	−4.4 (4)
C19—N3—C12—N2	179.4 (2)	C19—C24—C23—C22	2.7 (4)
C19—N3—C12—S1	−0.6 (4)		

### Hydrogen-bond geometry (Å, º)

Cg3 is the centroid of the C13–C18 ring.

**Table:**

*D*—H···*A*	*D*—H	H···*A*	*D*···*A*	*D*—H···*A*
C7—H7···O3^i^	1.00 (2)	2.55 (2)	3.197 (3)	122 (1)
C9—H9···O2^ii^	0.97 (2)	2.58 (2)	3.400 (3)	142 (1)
C15—H15···O1^iii^	0.93	2.59	3.286 (3)	132
C3—H3···*Cg*3^iv^	0.93	2.80	3.560 (3)	140

Symmetry codes: (i) −*x*, *y*+1/2, −*z*+1/2; (ii) −*x*, *y*−1/2, −*z*+1/2; (iii) −*x*, *y*−3/2, −*z*+1/2; (iv) *x*, −*y*+1/2, *z*−3/2.
